# Protein modifications in hepatic ischemia-reperfusion injury: molecular mechanisms and targeted therapy

**DOI:** 10.3389/fimmu.2025.1553298

**Published:** 2025-04-11

**Authors:** Xiaohong Zhao, Qinyi Li, Xiaolong Zhu, Yuanyuan Jiao, Huan Yang, Jiao Feng

**Affiliations:** School of Pharmacy, Hangzhou Normal University, Hangzhou, Zhejiang, China

**Keywords:** liver transplantation, hepatic ischemia-reperfusion injury, post-translational modification, targeted therapy, inflammatory response

## Abstract

Ischemia-reperfusion injury refers to the damage that occurs when blood supply is restored to organs or tissues after a period of ischemia. This phenomenon is commonly observed in clinical contexts such as organ transplantation and cardiac arrest resuscitation. Among these, hepatic ischemia-reperfusion injury is a prevalent complication in liver transplantation, significantly impacting the functional recovery of the transplanted liver and potentially leading to primary graft dysfunction. With the growing demand for organ transplants and the limited availability of donor organs, effectively addressing hepatic ischemia-reperfusion injury is essential for enhancing transplantation success rates, minimizing complications, and improving graft survival. The pathogenesis of hepatic ischemia-reperfusion injury is multifaceted, involving factors such as oxidative stress and inflammatory responses. This article focuses on the role of protein post-translational modifications in hepatic ischemia-reperfusion injury, including phosphorylation, ubiquitination, acetylation, ADP-ribosylation, SUMOylation, crotonylation, palmitoylation, and S-nitrosylation. Initially, we examined the historical discovery of these protein post-translational modifications and subsequently investigated their impact on cellular signal transduction, enzymatic activity, protein stability, and protein-protein interactions. The emphasis of this study is on the pivotal role of protein post-translational modifications in the progression of hepatic ischemia-reperfusion injury and their potential as therapeutic targets. This study aims to conduct a comprehensive analysis of recent advancements in research on protein modifications in hepatic ischemia-reperfusion injury, investigate the underlying molecular mechanisms, and explore future research trajectories. Additionally, future research directions are proposed, including the exploration of interactions between various protein modifications, the identification of specific modification sites, and the development of drugs targeting these modifications. These efforts aim to deepen our understanding of protein post-translational modifications in hepatic ischemia-reperfusion injury and pave the way for innovative therapeutic interventions.

## 1 Introduction

Hepatic ischemia-reperfusion injury (HIRI) refers to liver cell and tissue damage caused by the restoration of blood flow after a period of ischemia. During ischemia, the blood flow is interrupted, and when it is restored, the liver cells and tissues suffer from hypoxia, oxidative stress, and immune responses. This phenomenon is commonly observed in liver surgeries, liver transplants, trauma, or the treatment of liver diseases. HIRI is an unavoidable complication that significantly increases the risk of primary graft dysfunction and acute/chronic rejection (1, 2).

**Figure 1:**
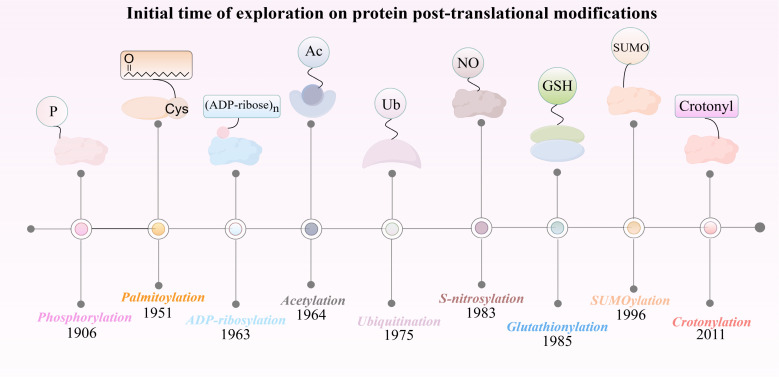
The discovery timeline of nine post-translational modifications closely associated with HIRI.

Its complex pathophysiology involves multiple mechanisms, including oxidative stress, inflammatory responses, cellular apoptosis, Kupffer cell activation, and neutrophil migration and activation (3–5). During ischemia, the interruption of blood flow leads to hypoxia and energy depletion, causing a decrease in intracellular ATP levels and loss of cellular function. Upon reperfusion, the sudden influx of oxygen generates reactive oxygen species (ROS) such as superoxide and hydroxyl radicals, which further damage cell membranes, DNA, and proteins. This process triggers an inflammatory response, as immune cells like neutrophils accumulate at the injury site, releasing cytokines and chemical mediators that exacerbate inflammation. Additionally, reperfusion compromises cell membrane integrity, disrupting the balance between intracellular and extracellular environments and leading to calcium overload—a significant rise in intracellular calcium ion concentration. Calcium overload not only threatens cell survival but also disrupts cellular energy metabolism by impairing ATPase activity, further weakening normal cellular functions (6). Mitochondrial function and structure are also severely affected, impairing energy production and exacerbating oxidative stress (7). The combined effects of oxidative stress and inflammation ultimately lead to cell death through apoptosis, necrosis, and autophagy, further aggravating liver damage (8). Both the differential expression of proteins and the dynamic regulation of their functions are essential factors that drive the development and manifestation of HIRI (**Figure 1**). Post-translational modifications (PTMs) refer to the covalent addition or removal of specific chemical groups to or from amino acid residues after protein synthesis. These modifications alter the physicochemical properties, three-dimensional structure, subcellular localization, and interactions of proteins with other biomolecules (9). To date, over 600 PTMs have been identified through advanced analytical techniques. Among these, enzyme-catalyzed modifications, such as phosphorylation, methylation, acetylation, sumoylation, ubiquitination, glycosylation, and palmitoylation, remain the most extensively studied (10).

In HIRI, PTMs play a crucial role in regulating cellular signaling, inflammatory responses, oxidative stress, and cell death. Recognizing the dysregulated role of PTMs in disease pathogenesis, several pharmacological agents targeting these modifications have shown enhanced therapeutic efficacy. For instance, inhibitors of histone modification, such as nicotinamide-an endogenous inhibitor of Sirtuin- modulate the activity of SIRT1, SIRT2, and SIRT3 (11). Similarly, histone deacetylase (HDAC) inhibitors, such as trichostatin A (TSA), vorinostat, and romidepsin (12), have demonstrated extensive clinical utility (13). These finding underscore the potential of pharmacological agents targeting PTMs as promising therapeutic strategies for wide range of diseases, including HIRI.

Research on PTMs associated with hepatic HIRI has been advancing rapidly. A detailed understanding of the molecular mechanisms underlying PTMs and their role in HIRI could offer critical insights for the development of preventive and therapeutic strategies. This review, therefore, aims to present a comprehensive analysis of recent progress in PTM research related to HIRI, explore the molecular mechanisms involved, and highlight potential directions for future research.

## 2 Functions and mechanisms of several key PTMs in HIRI

### 2.1 Phosphorylations

**Figure 2:**
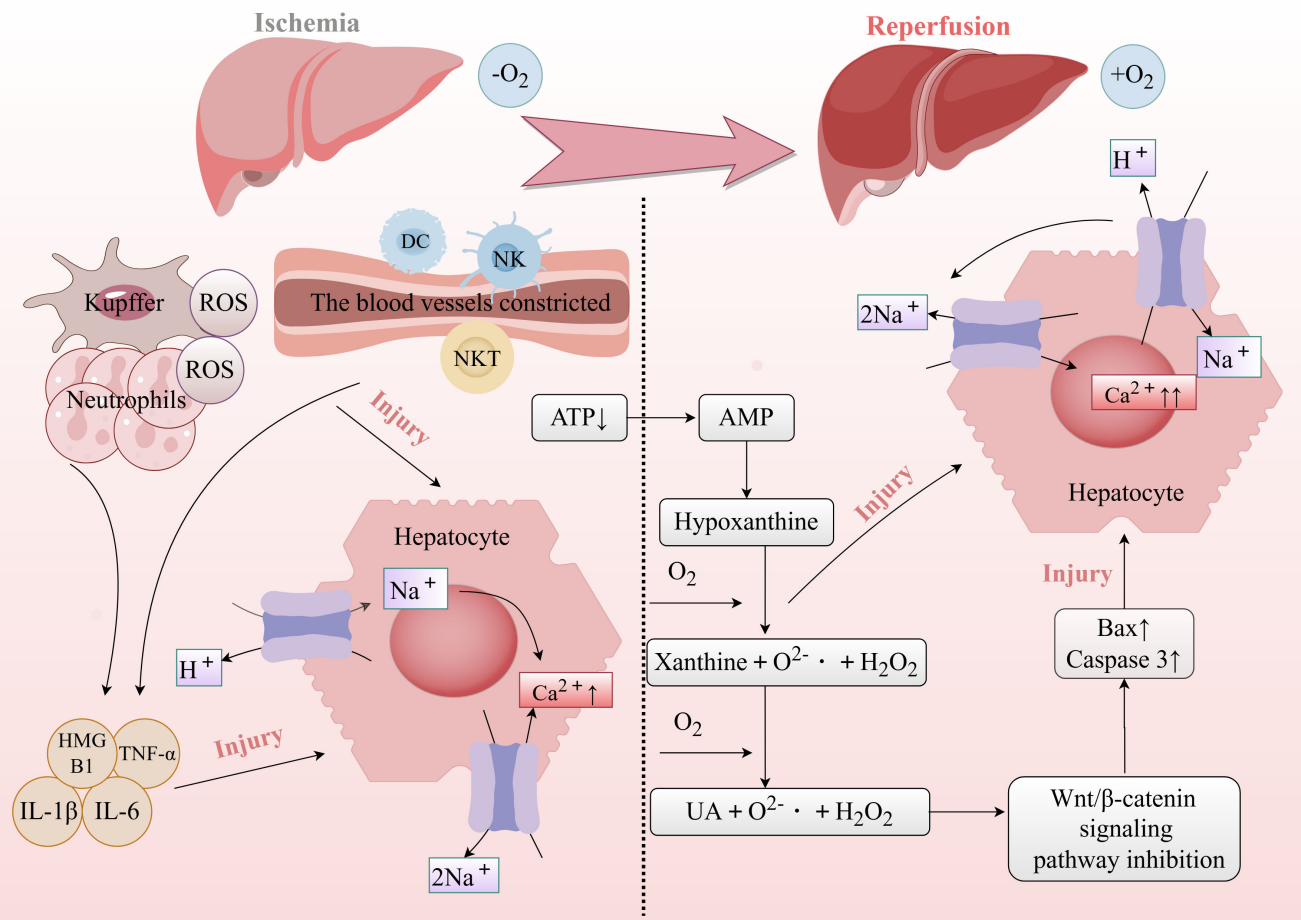
Mechanisms driving liver injury during the ischemia and reperfusion phases.

The discovery of phosphorylation can be traced back to the early 20th century, specifically in 1906 when P. A. Levene and C. L. Alsberg first reported on the phosphorylation of vitellin (**Figure 2**). This discovery marks the early study of protein phosphorylation (14). This discovery showed that protein molecules can reversibly attach phosphate groups through regulation by specific kinases and phosphatases. Protein phosphorylation can be categorized into four distinct types based on the specific amino acid residues that undergo phosphorylation: O-phosphate proteins, which are generated through the phosphorylation of hydroxyl-containing amino acids such as serine, threonine, or tyrosine (15); N-phosphate proteins, which result from the phosphorylation of arginine, lysine, or histidine (16); acylphosphate proteins, which are formed via the phosphorylation of aspartic acid or glutamic acid (17); and S-phosphate proteins, which are formed through the phosphorylation of cysteine residues (18). These classifications illustrate the mechanisms by which proteins modulate their intracellular function and activity via the phosphorylation of amino acid residues. Protein phosphorylation primarily influences cellular signaling, the regulation of enzyme activity, and protein-protein interactions. Indeed, it plays an integral role in cell signaling, facilitating cellular recognition and response to both external and internal stimuli, thereby modulating fundamental biological processes such as cell growth, division, and differentiation (19). Approximately 30% of the proteins encoded by the human genome are phosphorylated (10). Protein phosphorylation primarily affects cell signaling, enzyme activity, and protein interaction. In fact, phosphorylation plays an indispensable role in cellular signaling, facilitating the recognition and response of cells to both internal and external stimuli, thereby regulating fundamental biological processes such as cell growth, division, and differentiation (19). In terms of enzyme regulation, phosphorylation can enhance catalytic efficiency by inducing conformational changes or increasing substrate proximity, thereby altering enzyme activity (20). Interactions between proteins are crucial for cellular function, and phosphorylated proteins play a key role in forming multiprotein complexes that regulate these interactions (21).

Phosphorylation is a critical PTMs involved in HIRI, regulating key signaling pathways that influence cell survival, apoptosis, and inflammation. It plays a vital role in activating or inhibiting pathways such as MAPK and PI3K/Akt, modulating apoptosis-related proteins like Bcl-2 and Bad, and promoting the NF-κB signaling cascade. Through these mechanisms, phosphorylation contributes to the progression of liver injury by affecting cellular responses to oxidative stress and inflammation.

Dynamic phosphorylation of key proteins in inflammation-related signaling pathways, such as NF-κB, PI3K-AKT and cGAS-STING, plays a crucial role in the initiation and progression of HIRI. Specially, activation of the NF-κB signaling pathway exacerbates inflammatory responses and oxidative stress, further aggravating liver damage. Targeting phosphorylation in these pathways has been explored as a potential therapeutic strategy. For instance, pterostilbene, a natural compound, inhibits NF-κB phosphorylation and prevents its translocation to the nucleus, thereby alleviating inflammation and oxidative damage following HIRI.

**Figure 3:**
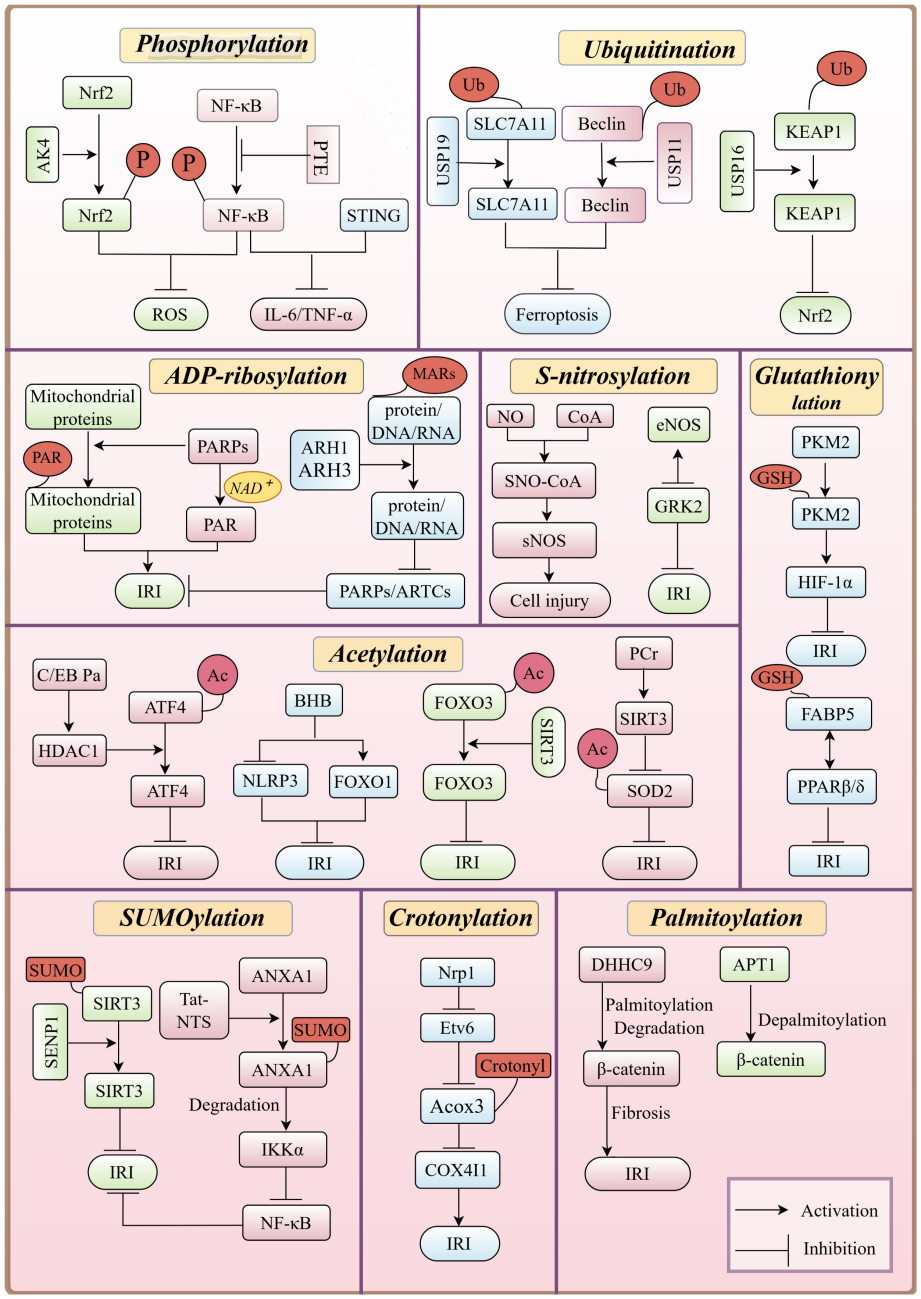
Overview of PTMs of proteins in HIRI, emphasizing their key roles, interactions, and regulation of signaling pathways, including the inhibition of NF-κB phosphorylation, which reduces inflammation during HIRI pathology.

Additionally, the cGAS-STING pathway is activated during IRI due to the release of mitochondrial DNA (mtDNA) and other damage-associated molecular patterns (DAMPs) from injured cells. This activation leads to STING dimerization and phosphorylation, which in turn promotes the synthesis of pro-inflammatory cytokines, including interferons (IFNs), IL-6, and TNF-α, exacerbating tissue damage. Inhibition or knockout of STING has been shown to reduce inflammation and apoptosis, highlighting STING phosphorylation as a therapeutic target for IRI (22) (**Figure 3**).

Phosphorylation also plays a key regulatory role in hepatocyte survival during IRI. The PI3K-AKT pathway, known for its protective effects against oxidative stress and apoptosis, is modulated by phosphorylation. Helium preconditioning-induced AKT phosphorylation has been shown to decrease hepatocellular injury and improve survival rate in experimental models (23). Similarly, Skimmianine alleviates HIRI by regulating inflammation, apoptosis, and oxidative stress through the phosphorylation of proteins related to the PI3K-AKT pathway (22).

Beyond inflammation and apoptosis, phosphorylation also affects the liver’s antioxidant defense mechanisms. During HIRI, p21-activated kinase 4 (PAK4) promotes the phosphorylation of Nrf2, a key transcription factor involved in the antioxidant stress response. This phosphorylation impairs Nrf2’s ability to combat oxidative stress, reducing hepatocyte resistance to IRI. Studies have shown that inhibiting PAK4 alleviates HIRI, while overexpression exacerbates the damage (24).

In addition to the pathways mentioned above, phosphorylation of other major proteins also influences HIRI. For example, CXCL16 silencing has been shown to alleviate HIRI during liver transplantation by inhibiting p38 phosphorylation, further underscoring the importance of phosphorylation in the regulation of liver injury (25).

In summary, phosphorylation plays a multifaceted role in HIRI by modulating inflammatory responses, oxidative stress, apoptosis, and antioxidant defenses. Targeting phosphorylation in key signaling pathways, such as NF-κB, PI3K-AKT, cGAS-STING, and Nrf2, presents a promising therapeutic strategy for mitigating liver damage following ischemia-reperfusion injury.

### 2.2 Ubiquitination

The history of protein ubiquitination modifications dates back to 1975, when Goldstein et al. serendipitously identified the ubiquitin molecule during their research on thymic hormones (**Figure 2**). They noted its ubiquitous presence across all tissues and cells, which led to its naming as “ubiquitin” (26). In 1978, Hershko and Ciechanover made significant progress by identifying an ATP-dependent proteolytic pathway. They achieved this by isolating and purifying phase-associated factors from reticulocyte extracts (27). By 1980, they demonstrated that APF-1, an ATP-dependent protein hydrolysis factor, was identical to ubiquitin. This discovery uncovered ubiquitin’s fundamental role in proteolysis (28).

Ubiquitination begins with ubiquitin-activating enzymes (E1) using energy from ATP hydrolysis to form a high-energy thioester bond between ubiquitin and a cysteine residue in E1 (29). The activated ubiquitin is then transferred to a ubiquitin-conjugating enzyme (E2) without requiring ATP (30, 31). Ubiquitin ligases (E3) facilitate the final step by transferring ubiquitin from the E2 enzyme to a specific sustrate protein, forming an isopeptide bond with a lysine residue or the N-terminus (31, 32). In human cells, the ubiquitination machinery consists of relatively few E1 enzymes, dozens of E2 enzymes, and over 600 E3 variants, ensuring remarkable substrate specificity (33).

There are eight types of ubiquitination modifications (M1, K6, K11, K27, K29, K33, K48, and K63) based on the ubiquitin attachment site. The most studied is K48-linked polyubiquitination, which regulates protein degradation via the ubiquitin-proteasome system (UPS) (34). Polyubiquitin chains, form through lysine residues like K48, enhance substrate recognition for proteasomal degradation (35). The 19S subunit disassembles ubiquitin chains and directs substances into the 20S catalytic core for degradation, while deubiquitinating enzymes (DUBs) recycle ubiquitin for reuse.

Ubiquitination plays a crucial role in regulating protein degradation, inflammatory signaling, and autophagy, making it a key process in HIRI. By modulating oxidative stress, inflammation, and cell survival, ubiquitination directly impacts the progression and severity of HIRI.

Ferroptosis, a major forms of hepatocyte injury in HIRI, is tightly regulated by ubiquitination. For example, USP19 removes K63-linked ubiquitin chain from SLC7A11, preventing its proteasomal degradation. Increased USP19 expression during IRI stabilizes SLC7A11, reduces ferroptosis, and alleviates liver injury (36). Conversely, USP11 removes ubiquitin chain from Beclin 1, preventing its degradation and promoting autophagy. This activation of autophagy enhances ferroptosis, leading to poor neurological recovery following IRI (37).

Inflammatory responses in HIRI are also significantly influenced by ubiquitination. USP16 removes K48-linked polyubiquitin chains from KEAP1, stabilizing it and promoting the ubiquitination and degradation of Nrf2. This inhibits Nrf2 signaling, reducing antioxidant gene expression and making hepatocytes more susceptible to oxidative stress damage. Therefore, targeting USP16/KEAP1 to increase the ubiquitination level of KEAP1 may serve as a potential therapeutic target to alleviate HIRI (38).

Additionally, hypoxia-induced activation of IL-6 signaling pathway is exacerbates inflammatory. USP15 deubiquitinates and stabilizes MeCP2, which inhibits IL-6 pathway activation and reduces inflammatory factor production, thereby attenuating the inflammatory response (39). Similarly, IRI activates the Toll-like receptor 4 (TLR4) signaling pathway, while hypothermic oxygenated perfusion inhibits tissue factor pathway inhibitor-2. This promotes the ubiquitination and degradation of Toll/interleukin-1 receptor domain-containing adapter protein, negatively regulating the TLR4/NF-κB-mediated inflammatory response (40).

Similarly, in IRI, apoptosis Signal-regulating Kinase 1(ASK1) is activated, triggering the downstream JNK/p38 signaling pathway, leading to apoptosis and inflammatory. Ring Finger Protein 5 (RNF5) ubiquitinates Phosphoglycerate Mutase 5 (PGAM5), promoting its degradation and inhibiting the activation of ASK1, thereby alleviating inflammatory damage (41).

In summary, ubiquitination can influence cell survival and death by regulating the stability of proteins related to ferroptosis, autophagy, inflammation, and apoptosis. These findings provide a theoretical foundation for developing new therapeutic strategies, as targeting these ubiquitinating and deubiquitinating enzymes may serve as potential therapeutic approaches to mitigate IRI.

### 2.3 Acetylation

Histone acetylation was first discovered in the 1960s as a mechanism regulating histone-DNA interactions and thereby influencing gene expression (42). In the 1980s, the NAD^+^-dependent deacetylase family, known as sirtuins was identified, with SIR2 linked to longevity in yeast (43, 44). By the 21st century, the roles of the mammalian sirtuins in cellular stress, metabolism, aging, stemness maintenance, chromatin remodeling, autophagy, and apoptosis were further elucidated (43). Acetylation is a PTM primarily occurring on the ϵ-amino group of lysine (K) residues, though it can also occur on tyrosine (Y), threonine (T), and serine (S). It is categorized into histone acetylation, non-histone acetylation, protein N-terminal acetylation, and lysine acetylation. Histone acetylation, mainly occurring on the N-terminal tails of histones H3 and H4, is the most prevalent, (45). Acetylation involves various modifying enzymes, including acetyltransferases (such as GNAT family, Myst family, and P300/CBP family) and deacetylases (the HDAC family and sirtuin family) (46). Typically, acetylation activates gene expression by reducing the affinity between DNA and histones, thus enhancing the accessibility of transcription factors and machinery. Conversely, deacetylation generally represses gene expression by increasing histone-DNA affinity (47).

During HIRI, pathways such as oxidative stress, inflammatory responses, and apoptosis contribute to hepatocyte damage and hinder tissue repair. Acetylation modifications, particularly these mediated by deacetylases such as HDAC and sirtuin family, play a crucial role in regulating these processes by modulating protein activity (48–50).

In terms of oxidative stress regulation, histone acetylation affects the expression of antioxidant-related genes. IRI promotes the acetylation of Nrf2, thereby inhibiting its activity. However, SIRT1 deacetylae Nrf2, enhancing its function and promoting the expression of antioxidant enzymes like SOD and HO-1, which help alleviate oxidative stress (51). Similarly, SIRT3 deacetylates superoxide dismutase 2 (SOD2), enhancing its ability to neutralize ROS. However, during IRI, increased acetylation of SOD2 and reduced SIRT3 activity impair antioxidant defense mechanisms. In this context, the use of phosphocreatine can upregulate SIRT3 expression, decrease acetylated-SOD2 accumulation, and restore its activity (52). Additionally, SIRT3 can deacetylatePRDX3, further reducing mitochondrial oxidative damage and cell apoptosis (53).

Non-histone acetylation also plays a key role in inflammatory regulation. SIRT1 deacetylates the p65 subunit of NF-κB, suppressing its activity and reducing the production of inflammatory factors, thereby mitigating inflammation during HIRI (54). Moreover, SIRT1 regulates apoptosis by deacetylating caspase proteins, inhibiting their activity, and modulating Bcl-2 family proteins. Specifically, it enhances Bcl-2’s anti-apoptotic function while suppressing the pro-apoptotic activity of Bax and Bad. Additionally, SRT1 deacetylates FOXO1/3, promoting the expression of autophagy-related genes such as Beclin-1 and LC3, thereby activating autophagy and reducing HIRI-induced damage (51).

Pharmacological interventions targeting acetylation modifications have shown therapeutic potential in HIRI. Berberine can act as an activator of SIRT1/FOXO3 pathway, demonstrating protective effects against oxidative stress and apoptosis. (55). Similarly, histone deacetylase inhibitors (HDACi), such as the endogenous HDACi β-hydroxybutyrate (BHB), alleviates IRI-related inflammation by upregulating FOXO1 and suppressing the NLRP3 inflammasome (56). Additionally, HDAC1-mediated deacetylation of ATF4 by C/EBPα suppresses endoplasmic reticulum stress, mitigating HIRI (57). HDAC1 also regulates hepatocyte differentiation and hepatic progenitor cell differentiation via Sox9b, Cdk8, Fbxw7, and Notch3 (58). With increasing insights into the role of deacetylases in disease, HDAC inhibitors have emerged as potential therapeutic agents. Several, including vorinostat, belinostat, panobinostat, and romidepsin, have already been approved by the FDA for clinical use (59, 60). Their regulatory mechanisms and therapeutic applications in liver diseases, including HIRI, remain an area of ongoing research (61, 62) (**Figure 3**).

### 2.4 ADP-ribosylation

The discovery of ADP-ribosylation dates back to the early 1960s, with Chambon making significant contributions to identifying this PTM (63) (**Figure 2**). ADP-ribosylation involves the transfer of ADP-ribose (ADPR) from NAD^+^ to target proteins, accompanied by the release of nicotinamide. This modification exists in two forms: mono-ADP-ribosylation and poly-ADP-ribosylation (64). ADP-ribosylation is a dynamic and reversible process regulated by two key enzyme families. ADP-ribosyltransferases (ARTs) covalently attach ADPR to proteins, influencing their function. Conversely, ADP-ribosylhydrolases (ARHs) remove these modifications, ensuring a balanced regulation of ADP-ribosylation in the body (65).

During IRI, ADP-ribosylation plays a central role in regulating apoptosis, inflammatory responses, and mitochondrial function. Poly(ADP-ribose) polymerase-1 (PARP-1), a key enzyme in this process, is involved in DNA repair, transcriptional regulation, and genomic stability. The interplay between ARTs (such as PARP-1) and ARHs maintains the homeostasis of ADP-ribosylation, significantly impacting the cellular response to IRI (66, 67).

During IRI, ADP-ribosylation plays a dual role, exerting either protective or detrimental effects depending on the level of activation. Moderate ADP-ribosylation can enhance cellular tolerance to damage by regulating PARP-1-mediated transcription factors, while excessive activation leads to cell death (68).

During IRI, cells initially experience oxidative stress, leading to increased generation of ROS. The rise of ROS activates PARPs, which use NAD^+^ to synthesize poly (ADP-ribose) (PAR), initiating ADP-ribosylation. The extent of PARP activation determines its effect on mitochondrial function and cell survival. At moderate levels, ADP-ribosylation supports DNA repair and cellular defense mechanisms. However, excessive activation depletes NAD^+^, leading to energy crisis and promoting cell death (69).

During IRI, DNA damage activates PARPs to facilitate repair through ADP-ribosylation (70). This leads to the depletion of NAD^+^ and the production of poly (ADP-ribose) (PAR), both of which play crucial roles in DNA repair through ADP-ribosylation (51). However, excessive activation can promote the accumulation of ADPR polymers in the cytoplasm and trigger the nuclear translocation of apoptosis-inducing factor (AIF) from mitochondria, thereby inducing apoptosis. Furthermore, PARP activation can result in the PAR of mitochondrial proteins, including those associated with the mitochondrial permeability transition pore (MPTP), such as cyclophilin D (CypD) and the translocator protein (TSPO), thereby affecting overall mitochondrial function (66, 71). In cardiac IRI, inhibiting PARP activity can reduce the size of myocardial infarction, suppress mitochondrial autophagy and apoptosis, and thus protect cardiomyocytes (72, 73). Given that PARP inhibitors minimize ADP-ribosylation, they are currently being investigated as potential therapeutic agents to mitigate IRI.

Furthermore, early studies have shown that ARH family proteins are closely associated with ADP-ribosylation. ARH1 and ARH3 specifically hydrolyze mono-ADP-ribosylation products (MARs) attached to proteins, DNA, or RNA. This hydrolysis reduces the extent of ADP-ribosylation modifications within cells (67). ARH family proteins can antagonize the functions of ARTs such as PARPs (74, 75) and ARTCs (76), which promote ADP-ribosylation by hydrolyzing ADP-ribosylation products. Therefore, ARH family proteins play a crucial role in maintaining the balance of intracellular ADP-ribosylation levels. In response to cellular stressors such as oxidative stress, ARH3 regulates PARP-1 activity and cellular stress responses by hydrolyzing ADPR at the termini of poly (ADPR) (PAR) chains. By hydrolyzing ADP-ribosylation products on mitochondria, ARH3 has the potential to influence mitochondrial function and membrane potential (ΔΨm), thereby affecting cellular energy metabolism and apoptosis (67). ADP-ribosylation plays multifaceted roles in IRI, including cellular stress, DNA repair, cell death, and inflammation. Regulating ADP-ribosylation in hepatocytes can protect cells from IRI-induced mitochondrial dysfunction and cell death, suggesting that modulating ADP-ribosylation may offer new therapeutic strategies for IRI-related diseases (**Figure 3**).

### 2.5 SUMOylation

Identified in 1996, SUMOylation is a PTM that involves the covalent attachment of small ubiquitin-like modifiers (SUMO) to target proteins (77) (**Figure 2**). This modification is widespread in eukaryotic cells and plays a crucial role in regulating protein stability, localization, and function. In higher eukaryotic cells, at least three SUMO proteins - SUMO1, SUMO2, SUMO3- have been characterized, along with six SUMO-specific proteases (SENP1-3, SENP5-7), which regulate the dynamic process of SUMO conjugation and deconjugation (78, 79).

SUMOylation is a dynamic and reversible biochemical process involving a coordinated series of enzymatic steps. It begins with the SUMO E1 activating enzyme, which uses ATP to activate SUMO proteins and transfer them to SUMO E2 conjugating enzymes (79). The E2 enzyme then facilitates the attachment of SUMO to a lysine residue on the target protein, a process further enhanced by SUMO E3 ligases. These ligases help ensure the efficient and specific conjugation of SUMO isoforms, regulating diverse cellular functions (80).

It is worth emphasizing that SUMOylation primarily affects IRI by influencing mitochondrial dynamics and inflammatory signaling pathways. Research indicates that SENP1 modulates the SUMOylation of SIRT3, which restores mitochondrial function, alleviates oxidative stress, and maintains the equilibrium between mitochondrial fusion and fission, thus preserving mitochondrial homeostasis (81). Additionally, Jing Huang et al. demonstrated that ALR (hepatopoietin) interacts with the transcription factor YY1 to inhibit the nuclear translocation of YY1, reducing the expression of UBA2, which in turn inhibits the SUMOylation of Drp1 (82). When the SUMOylation of Drp1 is reduced, its translocation to mitochondria is decreased, thereby attenuating mitochondrial fission and maintaining mitochondrial function (83).

On the other hand, the NF-κB pathway and the p38 MAPK signaling pathway are the major inflammation-related pathways affected by SUMOylation. In IRI, PIAS1 promotes the SUMOylation of NFATc1, thereby inhibiting its binding to HDAC1 and reducing the expression of HDAC1. The decreased expression of HDAC1 suppresses the expression of IRF-1, which in turn inhibits the activation of the p38 MAPK signaling pathway, and reducing inflammation and apoptosis (84). Additionally, the Tat-NTS peptide promotes the SUMOylation of ANXA1, inducing the transformation of microglia from a pro-inflammatory phenotype to an anti-inflammatory phenotype. This transformation facilitates the degradation of IKKα through selective autophagy mediated by NBR1 and inhibits the activation of the NF-κB pathway, leading to reduced expression and release of pro-inflammatory cytokines IL-1β and TNF-α, thus ameliorating IRI (85). Collectively, these studies demonstrate that SUMOylation plays an important role in alleviating IRI and provide a theoretical basis for the development of new therapeutic strategies (**Figure 3**).

### 2.6 Crotonylation

In 2011, Yingming Zhao’s research team identified crotonylation as a novel PTM affecting both histone and non-histone proteins (**Figure 2**). This modification involves the addition of a crotonyl group to lysine residues and associated with various cellular processes such as chromatin remodeling, cell metabolism, cell cycle regulation, and cellular reorganization (86).

Crotonylation is regulated by a variety of enzymes that control the synthesis, conversion, and clearance of the crotonyl group. The synthesis of the crotonyl group occurs through three pathways: Firstly, the production of crotonyl-CoA involves acetyl-CoA synthetase, which converts crotonic acid into crotonyl-CoA. Additionally, short-chain specific acyl-CoA dehydrogenases and acyl-CoA oxidase (ACOX3) play roles in the conversion of butyryl-CoA to crotonyl-CoA during fatty acid oxidation. Lastly, glutaryl-CoA dehydrogenase promotes the oxidation of glutaryl-CoA in the metabolic pathways of lysine, hydroxylysine, and tryptophan, thereby generating crotonyl-CoA. The conversion of the crotonyl group relies on a key enzyme, crotonyl-CoA hydratase (CDYL), which negatively regulates histone crotonylation by catalyzing the conversion of crotonyl-CoA to β-hydroxybutyryl-CoA, a process particularly crucial during spermatogenesis (87). HDACs, including HDAC1, HDAC2, HDAC3, and HDAC8, are responsible for removing crotonylation modifications from histones (88).

Notably, crotonylation plays a pivotal role in various cells by regulating protein functions and is closely associated with the pathogenesis of multiple diseases (89–91). The impact of crotonylation on the IRI process is first manifested in its regulation of mitochondrial function (92). For instance, the upregulation of neuropilin-1 (Nrp1) inhibits the transcription of Etv6, leading to a reduction in the expression of ACOX3, an enzyme involved in crotonyl-CoA synthesis, which, in turn, decreases the crotonylation of the mitochondrial protein Cox4i1, resulting in cell death through apoptosis (93). Additionally, during the early stages of IRI, the crotonylation levels of specific proteins such as isocitrate dehydrogenase (NAD^+^) 3 catalytic subunit alpha and the cytoskeletal protein Tropomyosin 1 (TPM1) significantly increase, which is crucial for maintaining mitochondrial and cytoskeletal protein functions (100). Given the central role of mitochondria in IRI, targeting crotonylation may help alleviate mitochondrial dysfunction and reduce the severity of IRI. Additionally, crotonylation influences signaling pathways related to cell death and apoptosis, such as regulating the expression of Bcl-2 family proteins, thereby potentially reducing IRI-induced cell death (94). Moreover, crotonylation can modulate inflammatory responses by affecting transcription factors critical to inflammation, such as NF-κB, offering therapeutic potential for controlling IRI (95) (**Figure 3**).

### 2.7 Palmitoylation

Although Japanese scientists first discovered palmitoylation modifications in the late 1950s, it initially received little attention (**Figure 2**). However, with advancements in detection techniques such as mass spectrometry, immunoprecipitation, acyl-biotin exchange, click chemistry, fluorescence resonance energy transfer (FRET), and metabolic labeling, the importance of palmitoylation in proteins has become increasingly evident (96).

Palmitoylation involves the attachment of a palmitoyl group to the cysteine residues of proteins, influencing their structure and function. This modification plays a critical role in regulating protein stability, subcellular localization, molecular interactions, and signal transduction. Palmitoylation occurs through two main mechanisms: enzyme-dependent and non-enzymatic regulation. Enzyme-dependent palmitoylation is catalyzed by palmitoyl acyltransferases (PATs), also known as zinc finger DHHC-type (ZDHHC) proteins. In mammals,23 ZDHHC enzymes have been identified, primarily localized in the Golgi apparatus and endoplasmic reticulum (ER), with some also found in the plasma membrane and mitochondria. The process involves two steps: self-palmitoylation of the enzyme to form an acyl-enzyme intermediate, followed by the transfer of the acyl group from CoA to the cysteine residue of the substrate protein. In contrast, non-enzymatic palmitoylation occurs independently of ZDHHCs and relies on acyl-CoA. Only a few specific proteins with strong acyl-CoA binding capacity can undergo autoacylation, and the underlying mechanisms remain unclear. Depalmitoylation, the reverse process, is mediated by thioesterases such as acyl protein thioesterases (APTs) and α/β-hydrolase domain-containing proteins (ABHDs), which remove the palmitoyl group from proteins and transfer it to the cytoplasm.

Palmitoylation plays a crucial role in IRI by influencing protein function, stability, and cellular signaling. This modification can inhibit protein activity by modifying thiol groups in oxidases and specific amino acid residues, leading to cross-linking of cytoplasmic and membrane proteins. Consequently, these modifications promote protein dimerization and disrupt normal cellular functions, contributing to the pathological changes observed in IRI. Due to its regulatory role, palmitoylation is emerging as a potential target for therapeutic intervention.

Mitochondrial dysfunction is a central feature of IRI, and palmitoylation significantly influences mitochondrial activity. Specifically, the S-palmitoylation of cysteine 202 (C202) on cyclophilin D (CypD) modulates the mitochondrial permeability transition pore (PTP), a key regulator of mitochondrial function during IRI. The dysregulation of PTP contributes to mitochondrial permeability changes, leading to cell death and exacerbating tissue damage (97).

Palmitoylation also plays a direct role in inflammatory cell death pathways, particularly pyroptosis. The S-palmitoylation of gasdermin D (GSDMD) enhance its pore-forming ability, facilitating pyroptosis and triggering inflammatory responses (98). Moreover, a positive feedback loop exists between S-palmitoylation and the production of ROS, further exacerbating cellular pyroptosis and inflammatory responses.

Palmitoylation exerts opposing effects on β-catenin stability, influencing fibrosis progression in IRI. During the later stages of IRI, the enzyme DHHC9 promotesβ-catenin degradation through palmitoylation, thereby alleviating fibrosis. Conversely, acyl protein thioesterase 1 (APT1) enhances β-catenin stability via depalmitoylation, facilitating its nuclear translocation and promoting fibrosis. The balance between these opposing mechanisms plays a critical role in determining the extent of fibrosis and organ dysfunction following IRI (99).

In addition, palmitoylation can modulate the release of cellular inflammatory factors, playing a role in the inflammatory response. The nucleotide-binding oligomerization domain-containing 2 (NOD2), also known as NLRC2, is a member of NLR family and is primarily expressed in immune and mucosal cells. ZDHHC5-mediated palmitoylation of NOD2 enhances the release of pro-inflammatory cytokines, including TNF-α, IL-1β, IL-6, as well as oxidative stress markers such as malondialdehyde (MDA) and myeloperoxidase (MPO), resulting in lung tissue damage (100, 101). Conversely, inhibition of S-palmitoylation using 2-bromopalmitate interferes with the autophagic degradation of NOD2, stabilizing the protein and modulating inflammatory responses (102). Beyond acute IRI, palmitoylation also plays a role in chronic inflammatory liver injury. The palmitoyltransferase ZDHHC3 exacerbates non-alcoholic fatty liver disease (NAFLD) by promoting the S-palmitoylation of inactive rhomboid protein 2, further highlighting the broader implications of palmitoylation in inflammatory conditions (103).

Currently, therapeutic strategies targeting the regulation of palmitoylation in IRI have been explored. Specifically, modulating the activity of palmitoyltransferases and acyl protein thioesterases by manipulating palmitoylation offers a novel approach to alleviating IRI (**Figure 3**). These strategies provide promising avenues for therapeutic interventions targeting molecular pathways involved in IRI and related conditions.

### 2.8 S-Nitrosation

S-nitrosylation was primarily discovered by Japanese scientists in 1983. S-nitrosylation is characterized by the covalent attachment of nitric oxide (NO) or its derivatives to the thiol groups of cysteine residues in proteins, forming S-nitrosothiols (SNOs) (**Figure 2**). This modification plays a significant role in cellular signal transduction, regulation of protein function, and influencing various physiological and pathological processes (104).

The historical background of S-nitrosylation is closely tied to the discovery and research of NO, a gaseous signaling molecule synthesized from L-arginine by nitric oxide synthase (NOS). Research progress has shown that NO can regulate proteins through S-nitrosylation, thereby influencing their structure and function. S-nitrosylation is modulated by the balance between NO production by NOS and the reduction of nitrosothiols by denitrosylases. Among denitrosylases, S-nitrosoglutathione reductase (GSNOR) plays a decisive role in regulating cellular homeostasis and human pathophysiology by modulating the bioactivity of NO (105, 106).

Nitrosylation has a significant connection with IRI, and it exerts either detrimental or protective effects in different organs suffering from IRI. The primary reason lies in the fact that during the cerebral IRI, the production of NO increases. Excessive NO can interact with the metabolic intermediate coenzyme A (CoA) to form S-nitrosocenzyme A (SNO-CoA), thereby affecting the nitrosylation of protein thiols. The resulting SNO may lead to impaired function, potentially causing cellular dysfunction and exacerbating cell damage and death (107, 108). However, in cardiac IRI, nitrosylation plays a protective role by maintaining nitro-oxidative balance and influencing the function of crucial proteins.

Previous studies have shown that in cerebral IRI models, inhibiting the levels of endogenous NO and S-nitrosylation of Stargazin can enhance synaptic remodeling, reduce neural damage, and decrease apoptosis. Additionally, suppressing the S-nitrosylation of receptor-interacting protein kinase 3 (RIP3) induced by cerebral ischemia also mitigates neuronal damage. These findings collectively suggest that modulating the S-nitrosylation of RIP3 and its downstream signaling pathways may be a promising therapeutic target for treating stroke (109, 110). In hippocampal Cornu Ammonis 1 (CA1) neurons damaged by cerebral IRI, inhibiting the S-nitrosylation of mitogen-activated protein kinase kinase 4 (MKK4) can alleviate neuronal degeneration and cell death, thereby providing neuroprotection (111).

Furthermore, S-nitrosylation plays a crucial role in cardiac protection by maintaining nitro-oxidative balance during IRI through the limitation of cysteine oxidation (111). Ischemic preconditioning promotes S-nitrosylation, thereby protecting protein cysteine residues from oxidation and facilitating cardiac recovery (112, 113). And the restoration of glyceraldehyde-3-Phosphate Dehydrogenase activity further confirms the protective function of S-nitrosylation in cardiac IRI (114). Besides, S-nitrosylation exerts cardioprotective effects by inhibiting the activity of G protein-coupled receptor kinase 2 (GRK2). Mechanistically, ischemia and reperfusion promote the interaction between endothelial NOS and GRK2, leading to the inhibition of GRK2 through S-nitrosylation, which affects the myocardial response to injury. GRK2-C340S knock-in mice exhibit increased sensitivity to IRI, revealing that the protective effects of NO bioactivity depend on the S-nitrosylation of GRK2 (115–117).

Currently, research on liver IRI is still limited. However, studies on IRI in other brain and heart suggest that S-nitrosylation holds multiple potential therapeutic benefits for IRI. Modulating the levels of S-nitrosylation and targeting specific sites may represent a novel strategy for treating IRI (**Figure 3**).

### 2.9 Glutathionylation

The discovery of glutathionylated proteins dates back to 1989 when Mustafa Aktan and his colleagues found that glutathione reacts with protein thiol groups to form these proteins (**Figure 2**). Glutathionylation is a PTM characterized by the formation of mixed disulfide bonds between glutathione and cysteine residues in proteins, driven by the active thiol group (-SH) of glutathione (118). This modification results in S-glutathionylated proteins.

In IRI, glutathionylation plays a crucial role in regulating ROS accumulation-related oxidative stress, influencing protein nuclear localization, and modulating inflammatory responses by protecting protein thiol groups from peroxidation. IRI triggers the production of ROS, leading to an imbalance in intracellular redox status. Under these conditions, ROS-sensitive pyruvate kinase M2 (PKM2) undergoes glutathionylation, causing its conversion from a tetrameric to a dimeric form and promoting its translocation into the nucleus. Within the nucleus, PKM2 can act as a cofactor, enhancing the expression of hypoxia-inducible factor 1α (HIF-1α)-dependent genes, and this process subsequently affects the stability and transcriptional activity of HIF-1α, thereby protecting against IRI (119, 120). Additionally, glutathionylation modulates the fatty acid-binding capacity and nuclear translocation of fatty acid-binding protein 5 (FABP5), while enhancing its interaction with peroxisome proliferator-activated receptor β/δ, suppressing the inflammatory response in macrophages. This elucidates a novel molecular mechanism of cellular protection under oxidative stress (121). Furthermore, reduced expression of the glutathionylation enzyme Glutaredoxin 1 (Grx1) is associated with increased severity of acute lung injury. Notably, Grx1 knockout mice exhibit protection against hyperoxia or LPS-induced acute lung injury, validating the anti-inflammatory and protective functions of glutathionylation in macrophages.

In summary, glutathionylation plays a role in antioxidant defense, protein function regulation, nuclear transport, cell signaling pathway modulation, anti-inflammatory effects, and mitochondrial function protection by regulating the glutathione and thioredoxin systems (122). These findings provide significant scientific insights for developing new strategies to mitigate IRI (**Figure 3**).

## 3 Techniques for analyzing post-translational modifications of proteins

PTMs are key mechanisms for regulating protein function, encompassing various types such as phosphorylation, acetylation, and ubiquitination. Traditional methods, including immunoprecipitation, Western blotting, and mass spectrometry, can identify specific modifications but are limited in efficiency. Modern high-throughput screening technologies, particularly mass spectrometry techniques (such as data-independent acquisition [DIA] and 4D-DIA) and quantitative mass spectrometry methods (such as TMT, iTRAQ, and LFQ), have significantly enhanced the detection coverage and quantitative accuracy of PTMs (123). For example, in studies of HIRI, DIA mass spectrometry has enabled researchers to rapidly identify and quantify a large number of modification sites, revealing the critical role of DExH-box helicase 58 (DHX58) in regulating ferroptosis in hepatocytes (124). Moreover, enrichment techniques (such as TiO_2_ enrichment of phosphorylated peptides and antibody enrichment of ubiquitinated proteins) and proximity labeling technologies (such as BioID and TurboID) have further improved research efficiency, allowing dynamic analysis of protein interaction networks in living cells. For instance, high-throughput screening technologies using biotin labeling and streptavidin bead capture, combined with mass spectrometry, can identify SUMOylation-modified proteins and their interaction networks, providing a new perspective for the study of fibrotic diseases (125).

## 4 Current research gaps and future research directions

Despite significant progress in recent years regarding protein modification in IRI, numerous gaps and challenges remain to be addressed. During the IRI process, in addition to the PTMs of proteins in the main component hepatocytes, the liver microenvironment also involves changes in the PTMs of proteins in various immune cells and hepatic stellate cells. Currently, research in these areas is lacking. Moreover, various PTMs of proteins are dynamically regulated, and as the disease progresses, the impact of a certain protein PTMs on IRI may yield completely opposite results. And there is a lack of specific markers for dynamically monitoring the IRI state of proteins in the body, which hinders the precise use of drugs targeting protein PTMs. Various PTMs of proteins also influence each other. When one type of protein PTMs is intervened, it may affect the state of other types of protein translations, ultimately failing to achieve the desired effect of the intervention measures. All of these factors will make it difficult to treat IRI by targeting protein PTMs. For these problems, future research should focus on several key areas: First of all, more research should be focused on the microenvironmental cells of the liver, research on hepatocytes alone is not enough, and the microenvironmental immune cells and fibroblasts need to be explored for their inflammatory response, repair contribution, and cellular communication in HIRI. Secondly, given the dynamic nature of IRI, where protein modification levels may fluctuate over time, future studies should utilize dynamic proteomics approaches to monitor these temporal changes. This approach could assist in elucidating the mechanisms of action of protein modifications at various stages of IRI. Moreover, interactions among various protein modification modes in IRI warrant further investigation. It is plausible that these modifications interact with one another. Hence, investigating these interactions and elucidating the regulatory mechanisms between different protein modifications could unveil the intricate regulatory network underlying IRI. Furthermore, future research could employ high-throughput technologies, such as mass spectrometry, to identify specific protein modification sites associated with IRI and analyze the functions of these sites through gene mutation and knockout techniques, thereby providing precise therapeutic targets for IRI. Finally, considering the interconnections and interactions between different protein PTMs, when targeting protein PTMs for the treatment of HIRI, it is preferable to target different protein PTMs in combination, and this will enhance drug efficacy.

In short, the development of therapeutics aimed at modulating protein modifications represents an emerging avenue in the treatment of IRI. Future research should employ high-throughput drug screening methods to identify compounds that specifically alter protein modification levels, followed by rigorous validation of their efficacy and safety through both *in vitro* and *in vivo* studies. Lastly, the translation of foundational research findings into clinical applications remains the primary objective in IRI research.

## 5 Conclusions

In the present review, starting from the discovery of various protein PTMs (**Figure 2**), we summarize and analyze the regulatory mechanism of each protein PTM in HIRI and drug development on the basis of the pathological and physiological mechanism of HIRI (**Figure 1**), And we revealed that HIRI is a complex pathological process wherein PTMs play a vital role. The dynamic nature of PTMs is fundamental for hepatocyte function and survival, influencing the cellular response to ischemic and reperfusion stress by modulating protein activity, stability, and interactions (**Figure 3**). Moreover, pharmacological interventions aimed at specific PTMs, including activators, inhibitors, or mimetics, may represent a viable strategy for the treatment of HIRI (**Table 1**). Investigating PTMs in HIRI presents a promising research avenue. This field holds potential for yielding significant insights into disease mechanisms and could provide essential information for developing new therapeutic strategies.

**Table 1:** PTM-related proteins and targets during HIRI: a mechanism summary.

Modification Type	Targets/Gene	Intervention Pathway	References
Phosphorylation	Pterostilbene	Inhibiting NF-κB Phosphorylation	(21)
	Helium	Inducing AKT Phosphorylation	(23)
	Skimmianine	Regulating phosphorylation of proteins related to the PI3K-AKT pathway	(22)
Ubiquitylation	USP16	Removes K48-linked polyubiquitin chains from KEAP1	(38)
	USP15	Deubiquitinates and stabilizes MeCP2	(42)
	RNF5	Ubiquitinating PGAM5	(44)
Acetylation	SIRT3	activates SOD2 through deacetylation	(52)
	SIRT3	SIRT3 enhance the activity of PRDX3 through deacetylation	(52)
	SIRT1	Deacetylates the p65 subunit of NF-κB	(52, 54)
	SIRT1	Enhance the activity of FOXO1/3 through deacetylation	(52)
	β-hydroxybutyrate	Upregulating FOXO1 and inhibiting the NLRP3	(59)
	HDAC1	Deacetylation of ATF4	(60)
ADP-ribosylation	PARP inhibitors	PARP mediated DNA damage and repair	(73)
	ARHfamily proteins	hydrolysing ADP-ribosylation products	(70)
SUMOylation	SENP1	maintains mitochondrial homeostasis	(84)
	PIAS1	SUMO mediates degradation of NFATc1	(87)
Crotonylation	Nrp1	Decreases the crotonylation of Cox4i1	(96)
	TPM1	Maintaining mitochondrial and cytoskeletal protein functions	(97)
	cyclophilin D	activity of the mitochondrial permeabilitytransition pore (PTP)	(100)
	GSDMD	Enhancing pore-forming ability and initiating pyroptosis	(101)
	NOD2	contributes to the release of TNF-α, IL-β, IL-6, MDA and MPO,	(103, 104)
Nitrosation	MKK4	protein kinase kinase 4/7 (MKK4/7)-JN signaling module	(114)
	GRK2	S-nitrosylation dependented NO bioactivity	(109, 120)
Glutathionylation	PKM2	HIF-1a	(122, 123)
